# Pulmonary Embolism Presenting as ST-Elevation Myocardial Infarction: A Report of Two Cases

**DOI:** 10.7759/cureus.61838

**Published:** 2024-06-06

**Authors:** Anthony J Dina, Sherell Hicks

**Affiliations:** 1 Emergency Medicine, University of Alabama at Birmingham School of Medicine, Birmingham, USA

**Keywords:** case report, ekg abnormalities, ultrasound imaging, st-elevation myocardial infarction (stemi), pulmonary embolism

## Abstract

Pulmonary embolism (PE) is often underrecognized due to its ability to mimic other conditions; however, ultrasound can provide diagnostic clues to aid in the diagnosis of PE. We describe two patients who presented with symptoms suggestive of cardiac ischemia and had electrocardiograms (EKGs) indicative of anteroseptal myocardial infarction. In both cases, cardiac point-of-care ultrasonography showed signs of large pulmonary emboli, which were then confirmed on computed tomography angiography of the chest. Both patients underwent successful aspiration thrombectomy with rapid resolution of cardiac dysfunction. Point-of-care ultrasonography should be used as an adjunct in patients presenting with anterior ischemia on EKG to evaluate for signs of PE.

## Introduction

ST-elevation in the setting of chest pain, shortness of breath, or syncope is commonly associated with myocardial infarction; however, several other conditions can mimic this presentation. Among them is pulmonary embolism (PE). PE is the third most common cause of acute cardiovascular disease after stroke and myocardial infarction, with an estimated annual incidence of 0.5-1 per 100,000 people [1]. Despite this, it is often unrecognized. Upon literature review, PE was unsuspected in 3268 (84%) of 3876 patients who had PE discovered at autopsy; even in patients with a large and potentially fatal PE, the majority (1902 of 2448, or 78%) were missed ante-mortem [2]. PE can be difficult to diagnose without high clinical suspicion due to its wide spectrum and often non-specific presentation, ranging from dyspnea to sudden death [3]. The challenge to diagnose PE is further exacerbated when the presenting electrocardiogram (EKG) is suggestive of ST-elevation myocardial infarction (STEMI) because the pressure to meet a 90-minute door-to-needle time is added to the immediate treatment considerations [4]. This is particularly poignant since inappropriate cardiac catheterization lab activation (CCL) can delay the time to appropriate diagnosis and treatment. We present two cases of PE presenting with anterior ST-elevation and T-wave inversions with concomitant inferior T-wave inversion, in which cardiac point-of-care ultrasonography (POCUS) before CCL transport resulted in a change in the care plan and the diagnosis of PE.

## Case presentation

Case one

**Figure 1:**
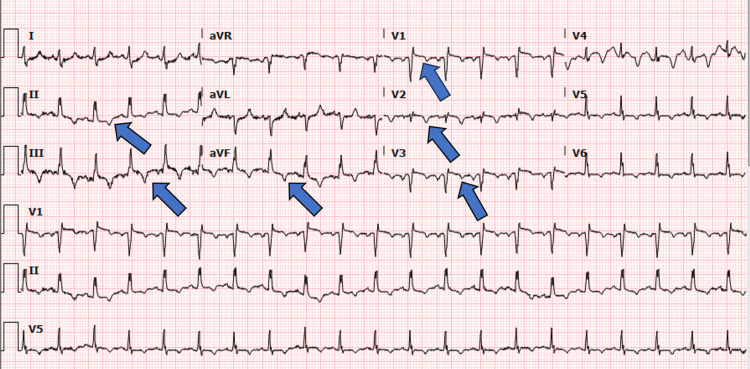
Electrocardiogram at presentation with ST-elevation and T-wave inversion in the anteroseptal leads with T-wave inversion in the inferior leads

A 57-year-old female presented to the emergency department (ED) via emergency medical services (EMS) for evaluation of chest pain, near-syncope, and shortness of breath, which began earlier the same day. The patient had no medical history, but her surgical history was notable for a left knee arthroscopy one month prior, which was complicated by a prolonged recovery period and immobilization. On arrival, the patient was noted to be cyanotic from the waist up, with her initial vital signs revealing a heart rate of 123 beats per minute (bpm), blood pressure of 99/73 mmHg, oxygen saturation of 100% on room air, respiratory rate of 28 breaths per minute (br/min), and a temperature of 97.8 °F. The arrival EKG revealed ST-elevation in the septal and anterior leads with deep T-wave inversion in the inferior leads concerning for septal and anterior STEMI (Figure 1).

**Figure 2:**
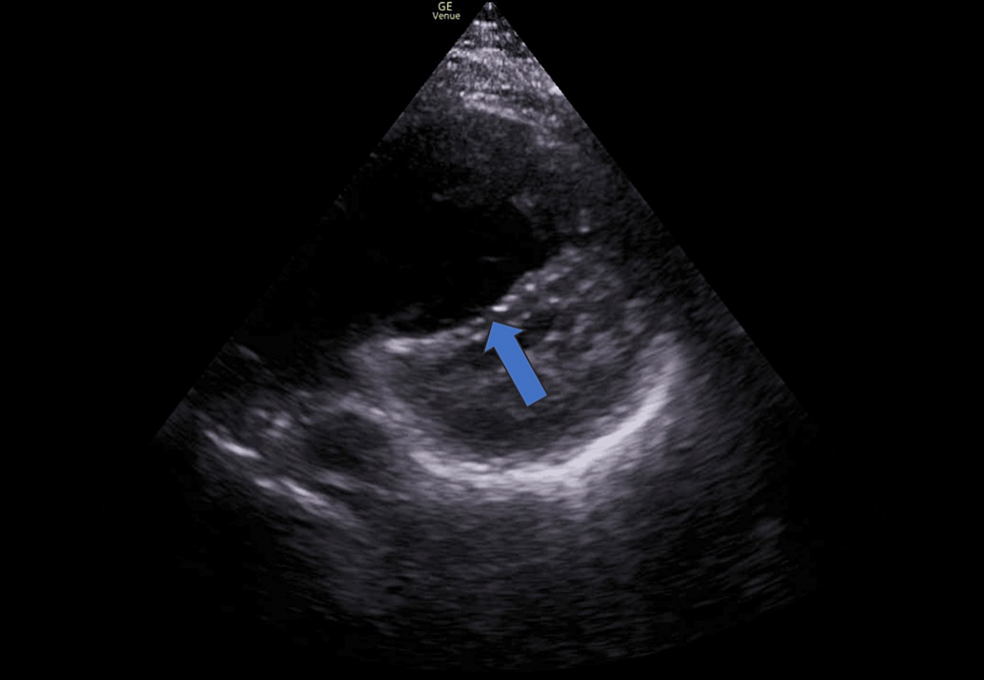
Point-of-care cardiac ultrasound showing right ventricular dilation and septal bowing

The cardiac catheterization lab was subsequently activated. Initial high-sensitivity troponin was elevated to 546 ng/L (reference range: 3-20 ng/L). During the cardiology evaluation, POCUS was performed and noted significant right ventricular (RV) dilation with septal bowing (Figure 2).

**Figure 3:**
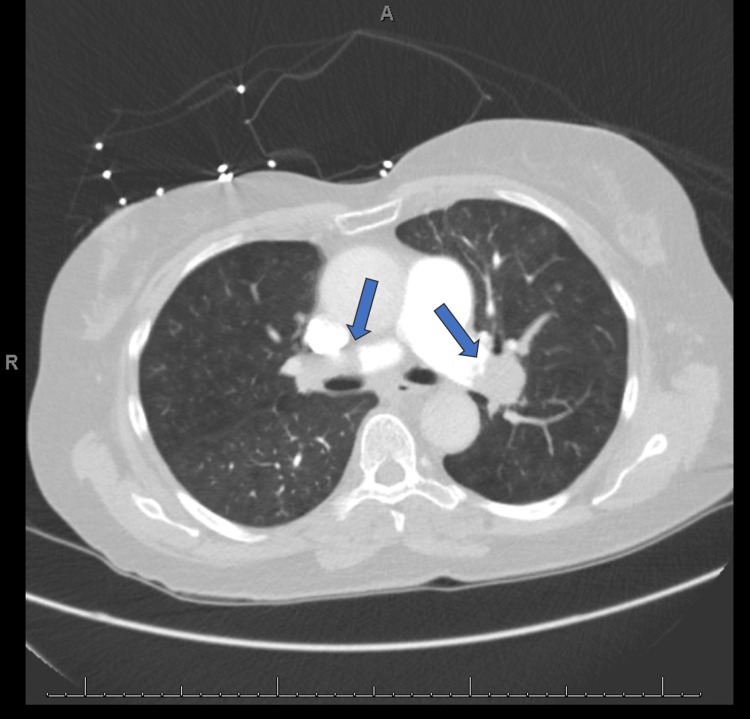
Bilateral central pulmonary emboli on the computed tomography angiography chest

The patient underwent an emergent computed tomography angiogram (CTA) of the chest, which was remarkable for large central PE bilaterally consistent with massive PE (Figure 3).

Due to concern for an impending hemodynamic collapse, the patient was immediately taken to the catheterization lab and underwent extracorporeal membrane oxygenation (ECMO) cannulation followed by mechanical thrombectomy with successful aspiration of the central PE. The patient was decannulated from ECMO the next day and transitioned from heparin drip to Eliquis. She was discharged on hospital day three in stable condition.

Case two

**Figure 4:**
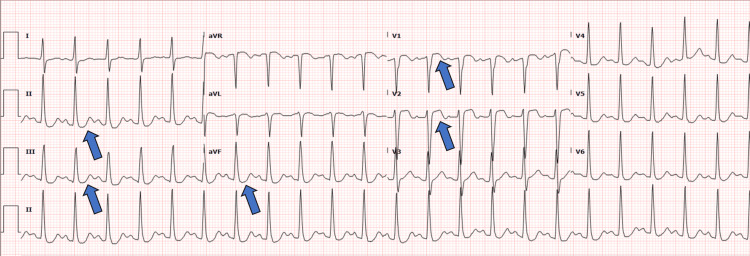
Electrocardiogram at presentation with ST-elevation in the septal leads and inferior ST-depression

A 64-year-old male presented to the ED via EMS for evaluation of a syncopal episode without prodromal symptoms while walking across the street. The pre-hospital EKG was concerning for anterior STEMI. On the initial evaluation, the patient reported the onset of shortness of breath one day prior to his arrival in addition to pre-syncope, but no chest pain at any point. His past medical history was significant for hypertension, chronic kidney disease, and diabetes mellitus. His initial vital signs revealed sinus tachycardia at a rate of 133 bpm, blood pressure of 129/89 mmHg, oxygen saturation of 100% on room air, respiratory rate of 22 br/min, and temperature of 98.9 °F. His physical exam was notable for moderate respiratory distress and diaphoresis. An EKG performed on arrival was notable for ST-elevation in septal leads with ST-depression in the inferior leads concerning for septal STEMI (Figure 4), and the cardiac catheterization lab was activated.

**Figure 5:**
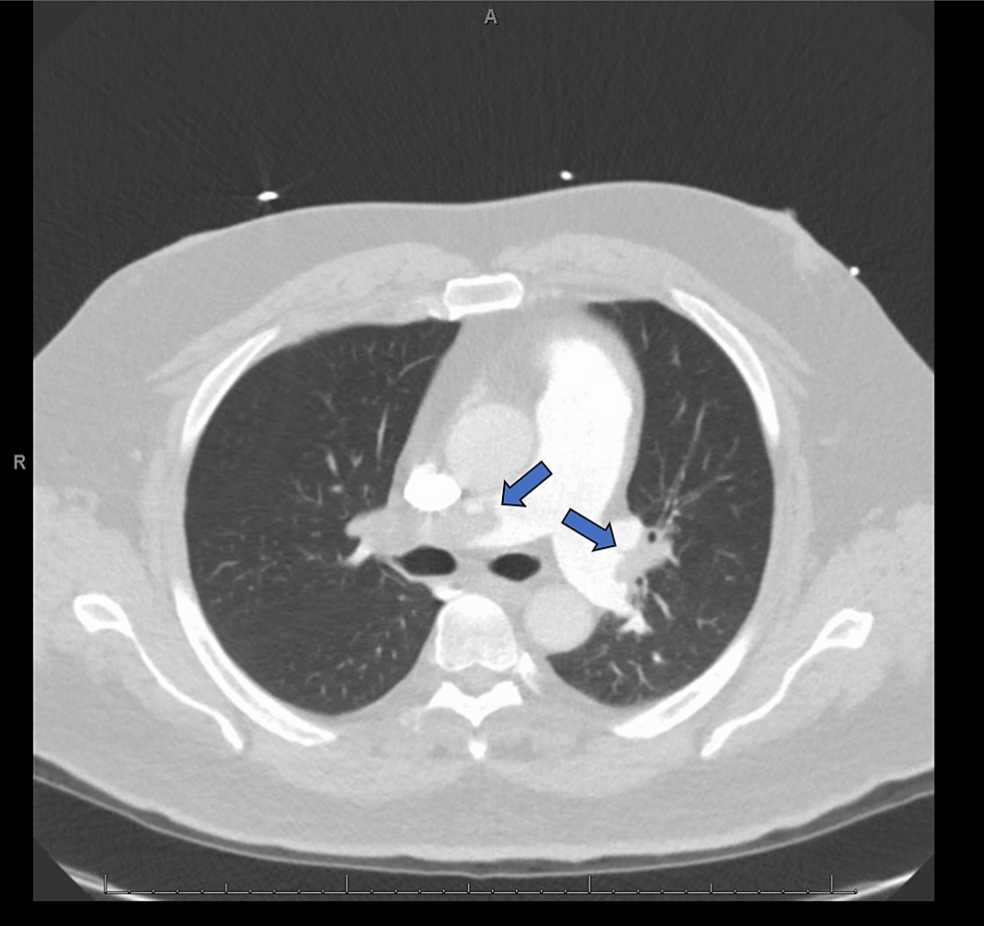
Bilateral central pulmonary emboli on the computed tomography angiography chest

On the initial cardiology evaluation, a cardiac POCUS was performed demonstrating RV dilation and septal bowing with global RV hypokinesis sparing the apex consistent with McConnell’s sign. Initial high-sensitivity troponin was obtained and elevated to 146 ng/L. The patient underwent emergent CTA, which revealed bilateral segmental and central pulmonary emboli (Figure 5).

While awaiting case review by the pulmonary embolism response team (PERT), the patient developed hypotension with blood pressure nadir of 82/60 mmHg, raising concern for developing obstructive shock due to massive PE. Levophed was started in consultation with the PERT physician, and the patient was taken for an emergent thrombectomy followed by the initiation of a heparin drip. The patient’s hospital course was otherwise unremarkable, and he was discharged home in stable condition on hospital day five after transitioning to Eliquis.

## Discussion

PE can cause a large number of EKG changes. The most common of these is sinus tachycardia; however, incomplete or complete right bundle branch block, atrial dysrhythmias, rightward QRS shift, S1Q3T3, acute pulmonary p-wave, T-wave inversion in the right precordial leads, and an anterior ischemic pattern described as inverted T waves in the precordium or ST-elevation have all been described in the literature [5-8]. The anterior ischemic pattern, in conjunction with initial symptoms that can mimic STEMI, presents a diagnostic conundrum to the emergency physician considering CCL activation. Fortunately, the EKG characteristics combined with cardiac POCUS may help guide decision-making.

Upon a literature review of PE presentation, an anterior ischemic pattern was noted to be the most sensitive (85%) and specific (81%) EKG finding for massive PE, which was defined as a Miller index of >50% (17/34) or mean pulmonary artery pressure (PAP) of >30 mmHg (with cardiac index maintained) [6]. Inverted T waves in leads III and V1 were also noted to be present in 88% of patients with PE, but only 1% of patients with acute coronary syndrome. These findings are indicative of rapid RV dilation and ischemia due to acute RV pressure overload, which can result in RV failure [9]. This is reflected in the American Heart Association (AHA) and European Society of Cardiology (ESC) guidelines, which define sub-massive (AHA) or intermediate-risk (ESC) PE as RV strain without hypotension on computed tomography scan or echocardiography or RV injury and pressure overload detected by an increase in troponins or brain natriuretic hormone. Massive (AHA) or high-risk (ESC) PE is defined as an RV strain with hypotension defined as a systolic blood pressure less than 90 mmHg, a drop of greater than 40 mmHg for at least 15 minutes, or a need for vasopressor support [10].

Unfortunately, consultative echocardiography is not readily available in the emergency room, but targeted POCUS can be utilized to evaluate for evidence of sub-massive or massive PE. The following findings on POCUS may suggest the presence of PE: abnormal tricuspid annular plane systolic excursion, McConnell’s sign (RV free wall hypokinesis with apical sparing), RV/LV ratio >1.0, D-sign (septal bowing) or paradoxical septal movement, thrombi in the right ventricle or pulmonary artery, or RV dilation (RV end-diastolic diameter >27) [11,12]. In our cases, POCUS before CCL transport identified signs of PE, which prompted an emergent CTA chest and the identification of life-threatening pulmonary emboli that would have otherwise been missed. We recommend the routine use of cardiac POCUS to evaluate for signs of PE in patients presenting with EKGs concerning for STEMI.

## Conclusions

PE is a common and potentially life-threatening diagnosis that can present as a STEMI mimic, causing diagnostic uncertainty. With the increasing availability of POCUS and the technical competency of emergency physicians, we propose the routine use of cardiac POCUS as an adjunct to evaluate for massive and sub-massive PE in patients presenting with anterior ischemic changes on the EKG before CCL transfer.
